# Less Conserved LRRs Is Important for BRI1 Folding

**DOI:** 10.3389/fpls.2019.00634

**Published:** 2019-05-21

**Authors:** Qiang Hou, Shehzadi Saima, Hong Ren, Khawar Ali, Chengke Bai, Guang Wu, Guishuang Li

**Affiliations:** ^1^ College of Life Sciences, Shaanxi Normal University, Xi’an, China; ^2^ Department of Plant and Microbial Biology, University of Minnesota, Saint Paul, MN, United States; ^3^ National Engineering Laboratory for Resource Developing of Endangered Chinese Crude Drugs in Northwest of China, Shaanxi Normal University, Xi’an, China

**Keywords:** Arabidopsis, BRI1, less conserved, LRRs, endoplasmic reticulum

## Abstract

Brassinosteroid insensitive 1 (BRI1) is a multidomain plant leucine-rich repeat receptor-like kinase (LRR-RLK), belongs to the LRR X subfamily. BRI1 perceives plant hormone brassinosteroids (BRs) through its extracellular domain that constitutes of LRRs interrupted by a 70 amino acid residue island domain (ID), which activates the kinase domain (KD) in its intracellular domain to trigger BR response. Thus, the KD and the ID of BRI1 are highly conserved and greatly contribute to BR functions. In fact, most *bri1* mutants are clustered in or surrounded around the ID and the KD. However, the role of the less conserved LRR domains, particularly the first few LRRs after the signal peptide, is elusive. Here, we report the identification of a loss-of-function mutant *bri1-235* that carries a mutation in the less conserved fourth LRR of BRI1 extracellular domain in Arabidopsis. This mutant had a base alteration from C to T, resulting in an amino acid substitution from serine to phenylalanine at the 156th position of BRI1. Compared with the wild-type plants, *bri1-235* exhibited round leaves, prolonged life span, shorter stature, and approximately normal fertility under light conditions. The *bri1-235* mutant was less sensitive to exogenous brassinolide under normal conditions. Importantly, both wild-type *BRI1* expression and a *sbi1* mutant that activates BRI1 rescued *bri1-235* and resembled the wild type. Furthermore, bri1-235 protein was localized in endoplasmic reticulum rather than plasma membrane, suggestive of a cause for reducing BR sensitive in *bri1-235*. Taken together, our findings provide an insight into the role of the less conserved LRRs of BRI1, shedding light on the role of LRRs in a variety of LRR-RLKs that control numerous processes of plant growth, development, and stress response.

## Introduction

In the complex and changing environment, plants have developed multiple strategies to sense various signals from their environment. These signals are first perceived at the cell surface and then transmitted to the cell’s interior. These events critically rely on cell-cell recognition and communication mediated by receptor-like kinases (RLKs). The structure organization of RLKs includes an extracellular domain, a single-pass transmembrane segment, and a cytoplasmic serine/threonine protein kinase domain (Shiu and Bleecker, 2001). Based on their different extracellular domains, RLKs can be divided into 13 subfamilies, of which the largest one is leucine-rich repeat (LRR) subfamily (Shiu and Bleecker, 2003). The LRRs in the extracellular domain of RLKs vary in number and distribution patterns, enabling them to have diverse functions. CLAVATA 1 (CLV1) with 21 LRRs controls meristem development (Clark et al., 1997). ERECTA (ER) and HAESA with 20 LRRs affect stomatal patterning and floral organ abscission, respectively (Stone et al., 1994; Jinn et al., 2000; Hunt and Gray, 2009). EXCESS MICROSPOROCYTES1 (EMS1) with 30 LRRs decides anther cell differentiation (Zhao et al., 2002). FLAGELLIN-SENSING 2 (FLS2) and EF-TU RECEPTOR (EFR), processing 28 LRRs and 21 LRRs, respectively, are involved in various responses to biotic stresses for innate defense in Arabidopsis (Gomez-Gomez et al., 2001; Zipfel et al., 2006). PHYTOSULFOKINE RECEPTOR 1 (PSKR1), with 21 LRRs interrupted by a 36-amino-acid island domain between 17th and 18th LRR, is involved in plant growth through elevating cell expansion and enhancing pathogen response (Matsubayashi et al., 2002; Amano et al., 2007; Mosher and Kemmerling, 2013). The co-receptor BRI1-ASSOSIATE KINASE1 (BAK1), with only five LRRs, directly associates with BRI1 in brassinosteroid signaling pathway and with FLS2 to mediate immune response (Nam and Li, 2002; Chinchilla et al., 2007). BAK1 also can interact with several other LRR-RLKs such as ER, EMS1, and PSKR1 (Zhao et al., 2002; Meng et al., 2015; Wang et al., 2015).

The crystal structure of a few LRR-RLKs have shown a right-handed superhelix of twisted LRRs (Di Matteo et al., 2003; Hothorn et al., 2011; She et al., 2011) or an anti-β-sheet (Wang et al., 2015). Variation in secondary structures on the convex side affects the curvature of the LRR domain, and this in turn permits interaction with an enormous diversity of ligands including hormone, peptide, and the entire protein. Many small signaling peptides have been identified, and typical examples are CLAVATA3 (CLV3) for CLV1 (Schoof et al., 2000), Phytosulfokine (PSK) for PSKR1 (Matsubayashi et al., 2002; Wang et al., 2015), Inflorescence deficient in abscission (IDA) for HAESA AND HAESA-LIKE2 (Butenko et al., 2003), and EPIDERMAL PATTERNING FACTORS (EPF) for ER (Shpak et al., 2005; Hara et al., 2007). The small protein, bacterial flagellin and TAPETUM DETERMINANT1 (TPD1) are perceived by FLS2 and EMS1, respectively (Gomez-Gomez and Boller, 2000; Yang et al., 2003). Among all LRR-RLKs in Arabidopsis, BRASSINOSTEROID INSENSITIVE 1 (BRI1) and BRI1-likes (BRLs) are the only examples that directly bind to phytohormone brassinosteroid (BR) (Cano-Delgado et al., 2004; Kinoshita et al., 2005). The newly formed BRI1-brassinolide (BL) surface can interact directly with extracellular residues of BAK1 *via* several hydrogen bonds (Santiago et al., 2013; Sun et al., 2013). The BRI1-BL-BAK1 complex can then initiate early BR signaling events and activate its downstream signaling cascade (Wang et al., 2008).

BRI1, with 25 LRRs and a 70-amino-acid island domain between 21st and 22nd LRR, regulates male fertility, flowering time, leaf senescence, vascular differentiation, dark-grown phenotype, and stress resistance (Clouse et al., 1996; Noguchi et al., 1999; Friedrichsen et al., 2000; Wang et al., 2001; Shang et al., 2011; Belkhadir et al., 2012; Gou et al., 2012). Three other BRI1 homologs, BRI1-LIKE 1 (BRL1), BRI1-LIKE 2 (BRL2), and BRI1-LIKE 3 (BRL3), were identified in Arabidopsis (Cano-Delgado et al., 2004; Zhou et al., 2004). Upon the expression of these homologs in Arabidopsis *bri1* mutants, their phenotypes were rescued by BRL1 and BRL3, but not BRL2, showing that BRL1 and BRL3 but not BRL2 can interact with BL of high affinity (Cano-Delgado et al., 2004; Kinoshita et al., 2005). Yet, BRL2 regulated vascular development (Ceserani et al., 2009). Based on these observations, BRI1 and its three homologs have specific functions in cell growth and vascular differentiation in Arabidopsis (Wang et al., 2001; Cano-Delgado et al., 2004; Kinoshita et al., 2005). Since the discovery of BRI1, more than 30 unique *bri1* mutants have been identified in Arabidopsis (Jiang et al., 2013). Their mutations are mainly clustered in the ID or the LRRs surrounding the ID in the extracellular region and the KD in the cytoplasmic region (Vert et al., 2005; Jiang et al., 2013). These mutants helped to determine the significance of the ID and KD of BRI1. However, the role of the less conserved LRR domains, particularly the first few LRRs after the signal peptide, remains unclear. The possible reason might be that this region is considered less important for BRI1 full function or may be neglected by researchers due to the lack of observable phenotype or systematic studies. Therefore, further studies are required to comprehensively understand the function of these less conserved LRR regions in different LRR-RLKs.

Here, we report the identification of a new mutation in the less conserved LRR regions of BRI1. This mutation significantly altered the growth and development of Arabidopsis plants. We described the isolation and characterization of this new *bri1* allele and named it *bri1-235*, which showed a weak morphological phenotype and a reduced sensitivity to BRs. The point mutation from C to T in the region encoding the 4th LRR of BRI1 resulted in the conversion of Ser to Phe at the 156th position, leading to mislocalization of BRI1 and less BR sensitivity. Our findings on *bri1-235* may provide insight to elucidate detailed functions of less conserved LRRs not only in BR receptors but also among numerous LRR-RLKs that control plant growth, development, and stress response.

## Materials and Methods

### Plant Materials and Growth Conditions

The ecotype Columbia (Col-0) of *Arabidopsis thaliana* was used as wild-type control. The *bri1-235* was selected in the Col-0 background by ethyl methane sulfonate (EMS)-mutagenesis. *bri1-5* mutant is in the Wassilewskija (Ws) background. The *sbi1* (suppressor of *bri1*) is an *A. thaliana bri1* suppressor mutant, which was identified from EMS-mutagenized *bri1-5* (Wu et al., 2011). *bri1-235 sbi1* was selected by crossing *bri1-235* and *sbi1* mutant. The seeds of Arabidopsis were surface-sterilized with 70% (v/v) ethanol for 3 min and 50% sodium hypochlorite for 8 min, followed by washing three times with sterilized water. After pre-incubated at 4°C in the dark for 3 days, the sterilized seeds were plated on 1/2 Murashige and Skoog (MS) medium containing 0.8% agar. After 7 days, seedlings were transferred to the moistened soil. Plants were grown under long daylight conditions (16-h light/8-h dark cycles) at 23°C.

### Isolation and Mapping of *bri1-235*

The seeds of ecotype Col-0 were mutagenized with 0.4% ethyl methanesulfonate (EMS) (Sigma). The homozygous M_0_ seeds grown in the greenhouse were pollinated with Landsberg erecta (Ler) pollens to propagate M_1_ seeds, which germinated and allowed self-pollination to generate a segregating M_2_ mapping population. The dwarf seedlings of M_2_ were selected and grown under normal light to produce M_3_ seeds, which were subsequently subjected to the second-step screening for those showing a normal etiolated phenotype when grown in the dark.

### Root and Hypocotyl Growth Assay

To determine the sensitivity of the plants to exogenous BR and BR biosynthesis inhibitor, the seeds of wild type, *bri1-235*, and transgenic plants were surface-sterilized, cold-stratified, germinated, and lined in petri plates containing 1/2 MS supplemented with 24-epibrassinolide (24-eBL, Sigma) at the 0, 0.1, 1, 10, 100, and 1,000 nM under light conditions or with propiconazole (PCZ) at the 0 and 5 μM under dark conditions. PCZ is a specific BR biosynthesis inhibitor in plants. PCZ binds to the BR biosynthesis enzyme CYP90D1, which is responsible for side chain C23 hydroxylation of BR, and inhibits the steps of BR biosynthesis from campestanol (CN) and teasterone (TE) (Hartwig et al., 2012; Oh et al., 2016). When the seedlings grew vertically in plate for 7 days, 20 seedlings were photographed and the hypocotyl length and root length were measured with Image J software. The measurement was repeated three times.

### Endo H Treatment

The leaves from 4-week-old soil-grown plants were homogenized in “Tris, NaCl, EDTA, and Triton X (TSEX) buffer” (50 mM Tris pH 8.0, 100 mM NaCl, 5 mM EDTA, 0.2% Triton X-100, 10% glycerol, and protease inhibitors). After boiling for 5 min, the leaf samples were centrifuged for 10 min at 10,000 g. After centrifugation, the protein extracts were denatured at 95°C for 10 min in denaturing buffer and incubated with or without 1,000 U of endoglycosidase H (Endo H) (New England Biolabs) in the G5 buffer for 1 h at 37°C. The immunoprecipitated proteins were separated by 7% SDS/PAGE and immunoblotted with anti-BRI1 antibody. The anti-BRI1 antibody was purchased from Agrisera. Horseradish peroxidase-linked anti-rabbit antibodies were used as secondary antibodies, and the signal was detected by Western blotting. The experiment was repeated three times.

### Confocal Microscopy

The localization patterns of BRI1-GFP, bri1-5-GFP, and bri1-235-GFP were examined by imaging the root tips of 5-day-old seedlings of *BRI1-GFP*, *bri1-5-GFP*, and *bri1-235-GFP bri1-235* double transgenic lines with a Leica TCSSP8 confocal microscope using 100× water immersion objective. GFP signal was acquired on conditions of excitation at 488 nm with emission at 505–530 nm.

### Generation of Transgenic Plants

Map-based cloning approach was used in cloning of *pBRI1:BRI1-GFP*, *pBRI1:bri1-235-GFP*, and *pBRI1:bri1-5-GFP* into expression. The full-length CDS fragment of BRI1 was amplified and cloned into a T-Vector PMD19. The cloned gene was sequence verified, digested, and cloned into the expression vector to get the construct *pBRI1:BRI1-GFP*, which was used for generating *BRI1-GFP* transgenic plants. The *BRI1-GFP* was transformed into *bri1-235* mutant *via* the *Agrobacterium tumefaciens* (GV3101)–mediated floral dip method (Clough and Bent, 1998). To confirm the transformation, the transformed seeds were germinated and selected on 1/2 MS medium containing kanamycin (50 mg L^−1^).

### Transcript Analysis by RT-PCR

The 2-week-old seedlings of Col-0, *bri1-235*, and *BRI1-GFP/bri1-235* grown on 1/2 MS plates were collected. Total RNAs were extracted from whole plant seedlings by using RNeasy Plant Mini Kit (Qiagen). The mRNA concentration was estimated by spectrophotometer. First-strand cDNA was synthesized from 2 mg of total RNA by utilizing M-MLV, a reverse transcriptase (Invitrogen), following the manufacturer’s instruction. The resulting cDNAs corresponding to 100 ng of total RNAs were amplified by using StepOnePlus Real-Time PCR System (Applied Biosystems) with gene-specific primers for *DWF4, CPD, BAS1*, and *ACT2* at an annealing temperature of 55°C for 30 cycles (Tanaka et al., 2005). Primers used for RT-PCR are given in the following paragraph.

*DWF4* (forward: 5′-CGAAGGAAGGCTCTTTGAATG-3′ and reverse: 5′-CTTCAACGGCTTTAGGGCAA-3′); *CPD* (forward: 5′-GTTCTTATCCTGCTTCCATTTG-3′ and reverse: 5′-AGCCACTCGTAGCGTCTCATT-3′); *BAS1* (forward: 5′-GTTCAGGACATTGTGGAGGAG-3′ and reverse: 5′-GGATAAAGCAACATAAGGACG-3′); and *ACT2* (forward: 5′-ACTCTCCCGCTATGTATGTCG-3′ and reverse: 5′-TGGACCTG CCTCATCATACTC-3′). RT-PCR experiment was repeated three times.

### Sequences Alignment and Phylogenetic Analysis

The amino acid sequences of BRI1, BRL1, BRL2, BRL3, and EMS1 of *Arabidopsis thaliana* were downloaded from the *Arabidopsis thaliana* TAIR^1^. The BRI1 protein sequences of *Brachypodium distachyon*, *Oryza sativa*, *Setaria italica*, *Sorghum bicolor, Zea mays, Solanum lycopersicum, Solanum tuberosum*, *Populus trichocarpa,* and *Phaseolus vulgaris* were retrieved by blast searches in the genome-wide sequenced plant databases from Phytozome v12.1^2^. EMS1 was defined as outgroup. The multiple sequence alignment was made by DNAman software. The full-length protein sequences were aligned with ClustalX and manually adjusted. The maximum likelihood tree was calculated with MEGA5.2.2 for 1,000 bootstrap repetitions. Branch lengths are proportional to the number of substitutions per 100 residues (indicated by the bar below the tree), and the branch point values indicate the percentage bootstrap support.

## Results

### Isolation and Characterization of a New *bri1* Allele, *bri1-235*

**Figure 1:**
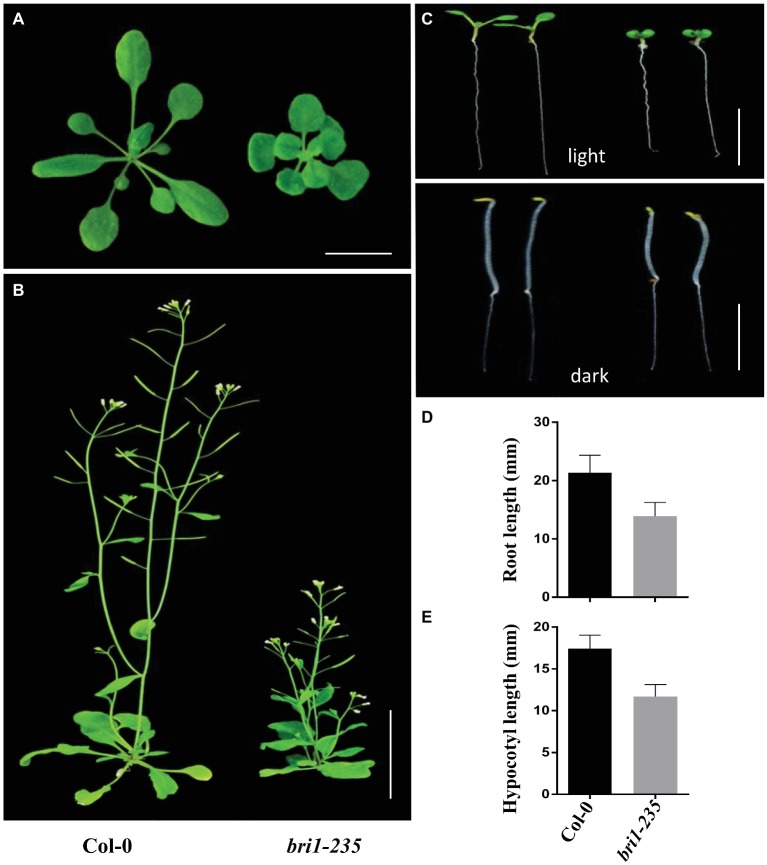
Phenotypes of *bri1-235* mutant. **(A)** One-month-old wild type and *bri1-23*5 grown under normal light at 23°C. Scale bar = 1 cm. **(B)** Two-month-old wild type and *bri1-235* grown under normal light at 23°C. Scale bar = 5 cm. **(C)** Seven-day-old wild-type and *bri1-235* seedlings grown on ½ MS plates under normal light or in the dark. Scale bar = 1 cm. **(D)** Quantitative measurements of root length of 7-day-old seedlings grown under normal light. **(E)** Quantitative measurements of hypocotyl length of 7-day-old seedlings grown in the dark. Data are presented the mean ± standard deviation from three independent biological replicates (*n* = 20 seedlings).

Using EMS mutagenesis and genetic screening, we isolated a weak morphological mutant when the mutants grew under normal light conditions. The mutants were dwarfed and displayed round leaves, short petioles, prolonged life span, reduced rosette size, and approximate normal fertility under the light conditions (Figures 1A,B). Compared to the wild-type Col-0, *bri1-235* mutants of Arabidopsis exhibited reduced root elongation under light conditions and reduced hypocotyl elongation under dark conditions (Figures 1C–E). These results suggest that the mutation in this mutant results in reduced BR response in Arabidopsis.

**Figure 2:**
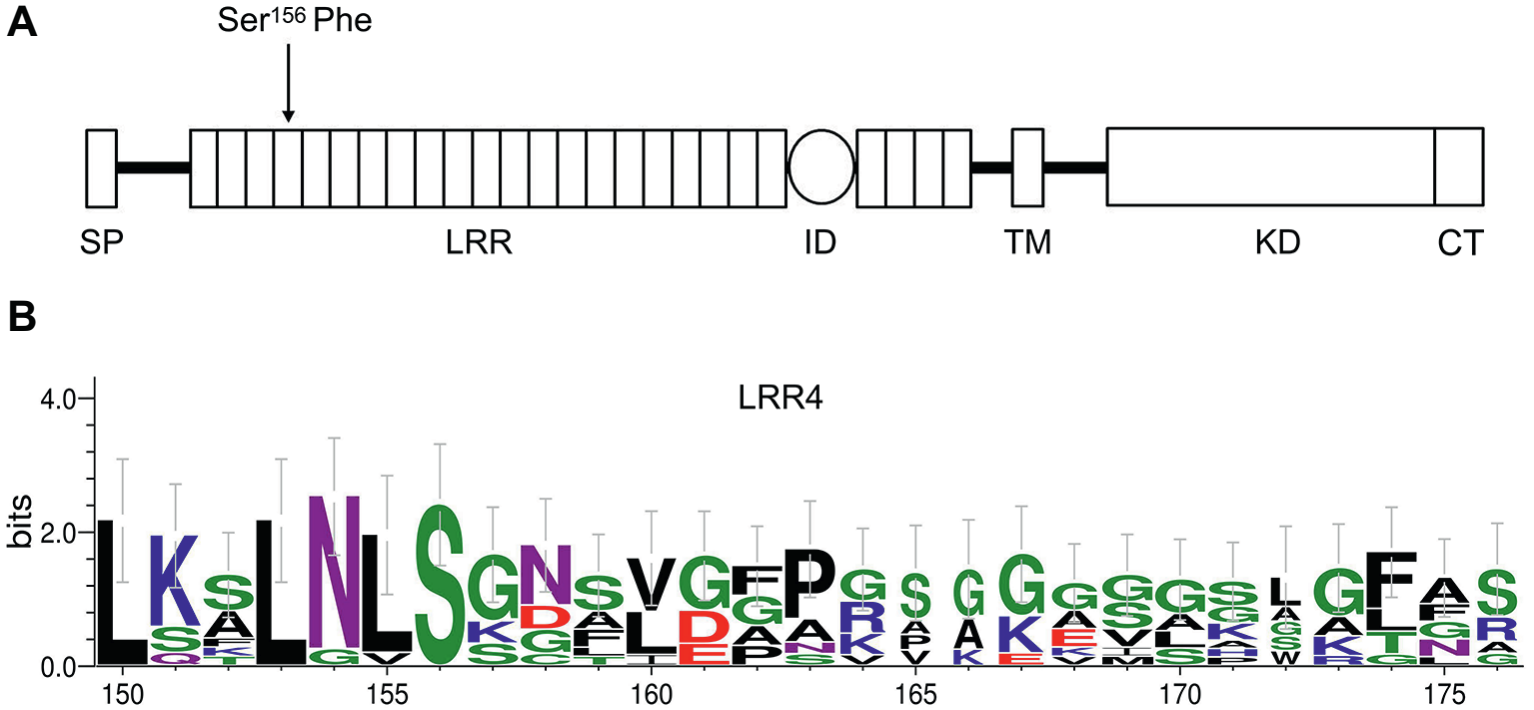
An amino acid substitution in *bri1-235* from Ser to Phe at the 156th position of BRI1. **(A)** The full-length BRI1 protein with a defined signal peptide (SP) domain, leucine-rich repeat (LRR) domain, island domain (ID), transmembrane domain (TM), kinase domain (KD), and C-terminal (CT). *bri1-235* has a point mutation in the region encoding the 4th LRR of BRI1, resulting in a change from Ser to Phe. **(B)** Sequence alignment of the 4th LRR among different species. S residue is highly conserved, and N at position 154 is the other highly conserved residue; however, other residues are less conversed among different species.

To identify the sequence alteration that resulted in *bri1-235* phenotype, we amplified the genomic region of *BRI1* using PCR and then performed DNA sequencing analysis on *bri1* allele, *bri1-235*. We found a single base alteration by comparing the genomic DNA sequences of the *bri1-235* mutant with the wild-type *BRI1*. The single base substitution from C to T in the 4th LRRs of *BRI1* resulting in an amino acid substitution from serine to phenylalanine at the 156th amino acid position of BRI1 in the *bri1-235* (Figure 2A). Sequence alignment of the 4th LRR showed that S residue in position 156 is highly conserved among different species. Apart from the serine at position 156, the other highly conserved residue in the 4th LRR is the asparagine at position 154, while most of its surrounding residues are not conversed among BRI1 in different species (Figure 2B).

### Most Amino Acids From the 1st to 10th LRR Are Variable Among BRI1 of Different Species

**Figure 3:**
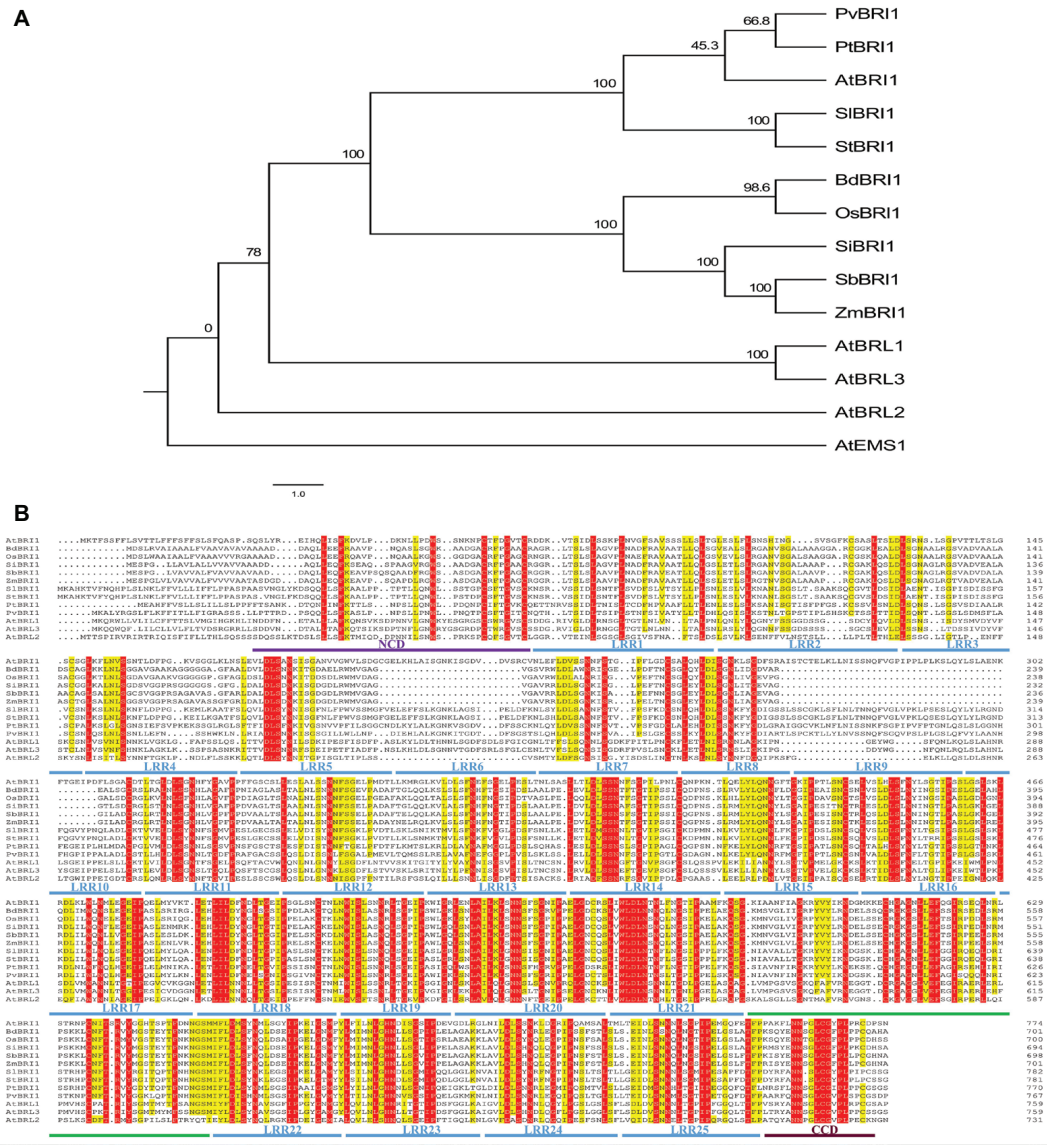
The phylogenetic relationship among BRI1’s homologs and sequence alignment of ectodomain BRI1 among different species. **(A)** The phylogenetic relationship among BRI1’s homologs. The full-length protein sequences were aligned with ClustalX and manually adjusted. The maximum likelihood tree was calculated with MEGA5.2.2 for 1,000 bootstrap repetitions. Branch lengths are proportional to the number of substitutions per 100 residues (indicated by the bar below the tree), and the branch point values indicate the percentage bootstrap support. **(B)** Conserved and similar residues are highlighted with red and yellow grounds, respectively. “NCD” and “CCD” represent the N-terminal and C-terminal capping domain, respectively. In **(A)** and **(B)**, “At,” “Bd,” “Os,” “Si,” “Zm,” “Sl,” “St,” “Pt,” and “Pv” represent *Arabidopsis thaliana*, *Brachypodium distachyon*, *Oryza sativa*, *Setaria italica*, *Zea mays*, *Solanum lycopersicum*, *Solanum tuberosum*, *Populus trichocarpa*, and *Phaseolus vulgaris*, respectively.

Among BRI1’s three homologs, BRL1 and BRL3 can also perceive and bind brassinosteroid; however, the ligand for BRL2 has not been found yet (Cano-Delgado et al., 2004). Phylogenetic analysis of BRI1 and its homologs in plants showed that BRI1 falls into the same clade with BRL1 and BRL3 but not with BRL2 (Figure 3A). Sequences of different plant species including five monocots (*Oryza sativa, Brachypodium distachyon, Setaria italic, Sorghum bicolor*, and *Zea mays*) and five dicots (*Arabidopsis thaliana, Solanum lycopersicum, Solanum tuberosum, Populus trichocarpa*, and *Phaseolus vulgaris*) were compared in similarity plots to study the conservation of the protein in this domain. Multiple sequence alignment of ectodomain BRI1 showed that the 11th–25th LRRs were highly conserved in the extracellular domains of BRI1 from different plant species. However, the 1st–10th LRR amino acid region of BRI1 extracellular domain was less conserved and highly variable (Figure 3B). In addition, there was insertion or deletion variation between the 1st and 10th LRR among different species. Similar alteration was found in between BRI1 and its homologs, BRL1, BRL3, and BRL2 from *Arabidopsis thaliana*.

### Both *BRI1* and *sbi1* Can Rescue *bri1-235* Phenotype

**Figure 4:**
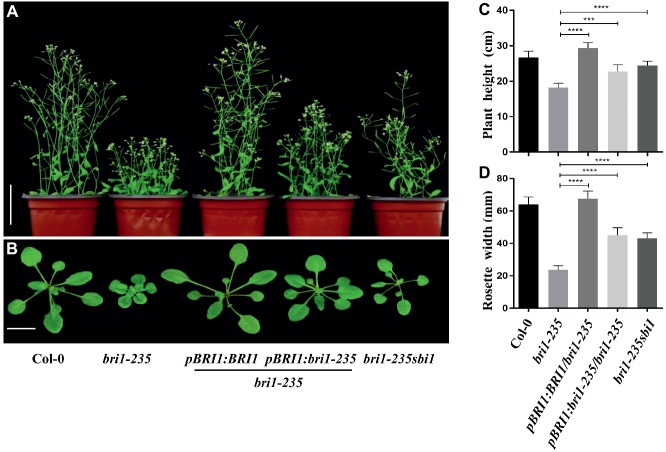
Phenotypes of *bri1-235* transgenic lines. **(A)** Two-month-old transgenic lines expressing *pBRI1:BRI1* and *pBRI1:bri1-235* in *bri1-235*, respectively. *bri1-235 sbi1* was produced by crossing *bri1-235* and *sbi1*. Scale bar = 1 cm. **(B)** One-month-old seedlings of the indicated genotypes. Scale bar = 5 cm. **(C)** Quantitative measurements of plant height grown under normal light. **(D)** Quantitative measurements of rosette width grown under normal light. Data are presented as the mean ± standard deviation from three independent biological replicates (*n* = 20 seedlings). ^***^ *p* < 0.001, ^****^ *p* < 0.0001 as one-way ANOVA with a Tukey’s test.

The phenotypic analyses of the *bri1-235* and the single-base exchange in the *BRI1* gene indicated that the mutant was caused by the loss-of-function mutation in the *BRI1* gene. To confirm our hypothesis, we generated transgenic plants that expressed *BRI1* or *bri1-235* by introducing a *BRI1* promoter-driven *pBRI1:BRI1-GFP* or *pBRI1:bri1-235-GFP* construct *via* the *Agrobacterium tumefaciens*–mediated floral dip method into *bri1-235*. Consistent with our prediction, the independent transgenic lines exhibited the phenotypes close to wild-type plants in the transgenics with *pBRI1:BRI1-GFP*, while overexpressing *bri1-235* can partially rescue *bri1-235* phenotypes. The *bri1-235* transgenic plants overexpressing *BRI1* showed the nearly normal overall growth patterns with elongated leaves and petiole length as well as total plant stature, similar to those observed in Col-0 as shown in Figures 4A,B. Simultaneously, we obtained *bri1-235 sbi1* double mutants by crossing *sbi1* to *bri1-235*. The *sbi1* is a loss of function allele of the gene *SBI1* encoding a leucine carboxylmethyltransferase that methylated protein phosphatase 2A and controlled its membrane-associated subcellular localization. Plant growth was promoted when *SBI1* was mutated in Arabidopsis. Moreover, *sbi1* regulates active BRI1 (Wu et al., 2011). Compared with *bri1-235*, *sbi1* partially rescued *bri1-235* phenotypes including plant height and rosette width (Figures 4C,D). These results indicate that *bri1-235* is a *bri1* allele with weak BR response and has kinase activity.

### The Sensitivity to BR is Reduced in *bri1-235*

**Figure 5:**
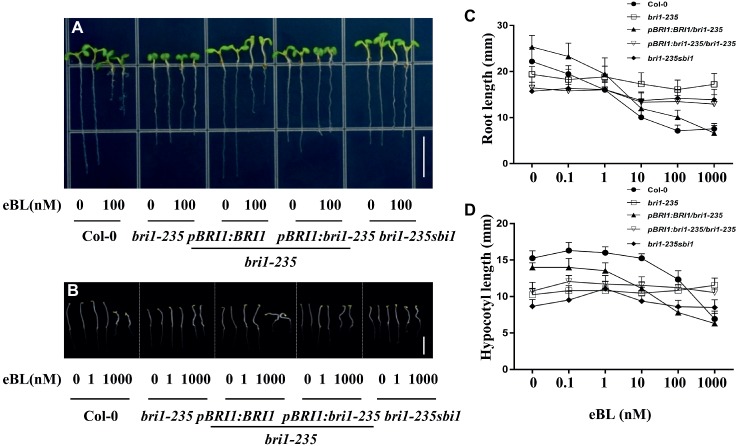
Sensitivity of *bri1-235* and its transgenic lines to exogenous eBL. **(A)** Root phenotype of 5-day-old seedlings under light conditions. Scale bar = 1 cm. **(B)** Hypocotyl phenotype of 5-day-old seedlings in the dark. Scale bar = 1 cm. **(C)** Quantitative measurements of root elongation under normal light. **(D)** Quantitative measurements of hypocotyl elongation in the dark. Data are presented as the mean ± standard deviation from three independent biological replicates (*n* = 20 seedlings).

As an essential plant hormone, BR can affect plant morphology at nanomolar level. To check sensitivity of *bri1-235* and its transgenic plants to BR and PCZ, seeds were grown in 1/2 MS medium with or without various concentrations of 24-eBL or PCZ. Root growth inhibition analysis showed that the root growth of wild type was sensitive to exogenous 24-eBL treatment in a concentration-dependent manner. However, the roots of *bri1-235* seedling were not significantly altered by the application of exogenous applied 24-eBL under light conditions (Figure 5). The transgenic lines, overexpressing *BRI1* or *bri1-235*, restored sensitivity to BR to a certain extent. *sbi1* can partially rescue the sensitivity to BR. With regard to the analyses of hypocotyl elongation in dark, the wild-type seedlings responded to exogenous 24-eBL in a concentration-dependent manner and showed reduced hypocotyl elongation, while *bri1-235* showed less sensitivity to BL (Figure 5).

**Figure 6:**
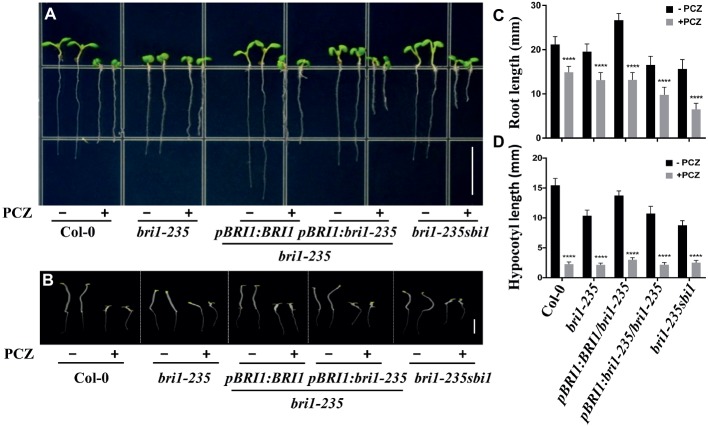
Sensitivity of *bri1-235* and its transgenic lines to exogenous PCZ. **(A)** Root phenotype of 5-day-old seedlings grown on ½ MS plates with or without 5 μM PCZ under light conditions. Scale bar = 1 cm. **(B)** Hypocotyl phenotype of 5-day-old seedlings grown on ½ MS plates with or without 5 μM PCZ in the dark. Scale bar = 1 cm. **(C)** Quantitative measurements of root elongation under normal light. **(D)** Quantitative measurements of hypocotyl elongation in the dark. Data are presented as the mean ± standard deviation from three independent biological replicates (*n* = 20 seedlings). ^****^ *p* < 0.0001 as one-way ANOVA with a Tukey’s test.

In addition, we also tested the sensitivity of this new *bri1* allele to PCZ, which is a specific BR biosynthetic inhibitor. As expected, the hypocotyl of this weak mutant and wild-type seedlings exhibited hypersensitivity to PCZ (Figure 6). Similar trend was observed for root length, but it was less sensitive to PCZ as compared to hypocotyl. These findings confirm that the sensitivity of *bri1-235* to BR or PCZ altered because of the substitution of serine with phenylalanine in the 4th LRR.

**Figure 7:**
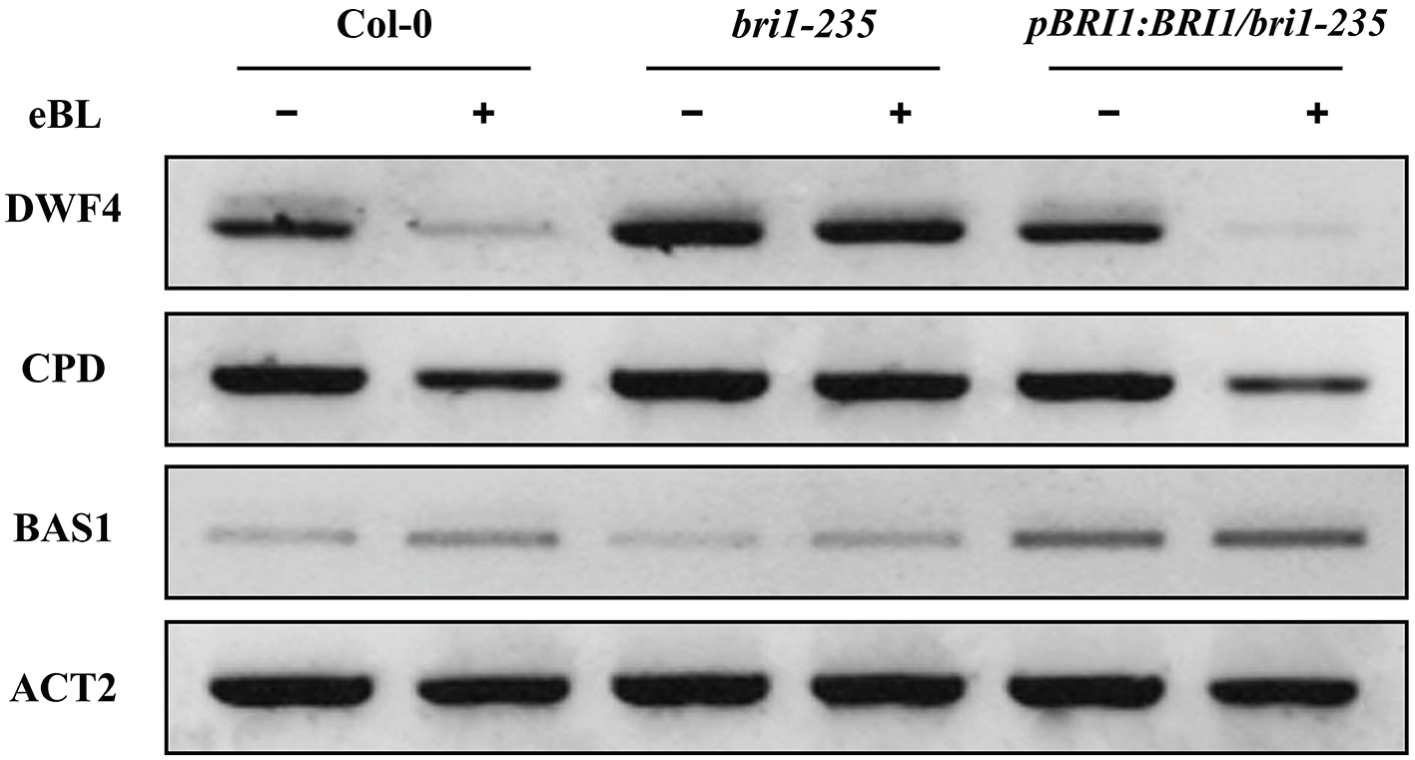
Expression levels of *DWF4*, *CPD,* and *BAS1* in wild type, *bri1-235*, and *pBRI1:BRI1/bri1-235* seedling grown on ½ MS medium without or with 100 nM eBL in the dark.

Several brassinosteroid response genes (i.e., *DWF4*, *CPD*, and *BAS1*) in the brassinosteroid signaling pathway are subjected to feedback regulation upon BL treatment. *DWF4* and *CPD* encode 22α-hydroxylase and C-3 oxidase, respectively, which are involved in BR biosynthesis and are negatively regulated by BRs (Mathur et al., 1998; Gonzalez-Gaitan, 2008). *BAS1* encodes a cytochrome P450 that is involved in BR degradation and is positively regulated by BRs (Turk et al., 2003). BR biosynthesis genes, *DWF4* and *CPD*, and BR inactivation genes *BAS1* are sensitive markers for enhanced BR signaling (Tanaka et al., 2005; Wang and Chory, 2006; Tang et al., 2008). To further test the altered response of *bri1-235* to exogenous 24-eBL, we checked the expression level of these BR responsive genes in the wild type, *bri1-235*, and the *pBRI1:BRI1-GFP/bri1-235* mutants under exogenous 24-eBL. The results show that the expression level of *DWF4* and *CPD* were strongly suppressed by 24-eBL treatment in wild-type but not in *bri1-235* mutants (Figure 7). Similar to wild type, the expression level of *DWF4* and *CPD* was strongly suppressed by 24-eBL in the *pBRI1:BRI1-GFP/bri1-235.* The expression of *BAS1* was significantly induced by exogenous 24-eBL in the wild-type plants, but slightly affected in *bri1-235* mutant. As expected, similar to the effect of BR on the wild plants, *BAS1* was significantly increased by exogenous 24-eBL in the *pBRI1:BRI1-GFP/bri1-235* (Figure 7).

### The bri1-235 Protein Is Localized in the Endoplasmic Reticulum

**Figure 8:**
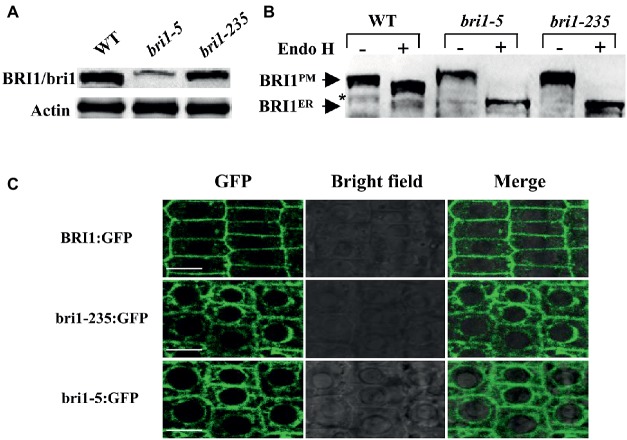
bri1-235 is retained in the endoplasmic reticulum (ER). **(A)** BRI1 and bri1 were detected by Western blotting. Actin served as the loading control. **(B)** Endo H assays of *bri1-5* and *bri1-235*. The arrows indicate the positions of bands representing BRI1 in the plasma membrane (BRI1^PM^) and in the ER (BRI1^ER^). The asterisk denotes a nonspecific band. **(C)** Confocal microscopic analysis of BRI1:GFP, bri1-235:GFP, and bri1-5:GFP. Fluorescence patterns of GFP fusion proteins, bright field, and the merged images of green and bright field signals in root tip cells of Arabidopsis are shown from left to right. Scale bar = 5 μm.

Mislocalization of cell surface receptors can lead to their malfunctions. To determine the mechanism of the loss function of *bri1-235*, we asked the localization of bri1-235 protein in *bri1-235*. The subcellular localization of bri1-235 was first analyzed by its sensitivity to Endo H that cleaves high mannose-type N-glycans of the endoplasmic reticulum (ER)-localized proteins, whereas it fails to cleave Golgi-processed complex glycans. Thus, Endo H treatment can distinguish the ER-localized BRI1 from the plasma membrane (PM)-localized BRI1. After the total proteins of the wild type, the *bri1-235* and *bri1-5* seedlings were treated with or without Endo H, the samples were subjected to gel electrophoresis followed by immunoblotting with anti-BRI1 antibody (Mora-Garcia et al., 2004). *bri1-5* carrying Cys69Tyr mutation is a proven *bri1* mutant whose bri1-5 protein is mainly located in ER (Hong et al., 2008). Here *bri1-5* is a positive control to support the ER localization of *bri1-235*. As shown in Figure 8A, the abundance of bri1-235 was reduced slightly than that of wild type. BRI1 in the wild type was located in the plasma membrane; however, the bri1-235 protein appeared completely sensitive to Endo H digestion, which indicates ER localization (Figure 8B). Confocal study revealed that bri1-235-GFP was localized in endoplasmic reticulum rather than plasma membrane as well (Figure 8C). *bri1-9* is another ER-retaining *bri1* mutant with Ser662Phe mutation in the 22nd LRR of BRI1 extracellular domain (Jin et al., 2007). Therefore, our results clearly demonstrate that *bri1-235* is a new mutant, similar to *bri1-5* or *bri1-9* mutants, in term of receptor’s mislocalization.

## Discussion

In the past years, molecular genetics studies have discovered and characterized many BR responsive genes that function in BR biosynthesis, signaling, and inactivation. These studies have provided insight into molecular mechanisms of BR action. *DWF4* and *CPD,* BR biosynthesis genes, were upregulated by exogenous BL in *bri1-235.* Meanwhile *BAS1,* a BR inactivation gene, was downregulated in *bri1-235* as compared to wild type (Figure 7). This indicates the feedback regulation of BR responsive genes to maintain BR homeostasis and regulate plant development.

The extracellular domains of LRR-RLKs are versatile and diverse, while its kinase domains are conversed in plant. The ID of BRI1 and the LRRs surrounding the ID, are responsible for perceiving BRs (Kinoshita et al., 2005). The crystal structure of BRI1 shows the ID of BRI1 folds back into the interior of the superhelix to bind BL. The superhelix makes the extensive polar and hydrophobic interaction with the 13th LRR–25th LRR (Hothorn et al., 2011; She et al., 2011). Such twisted assemblies of LRRs and superhelix also exist in the plant defence protein PGIP in *Phaseolus vulgaris* (Di Matteo et al., 2003). Therefore, twisted LRRs in brassinosteroid binding extend to the region from 13th LRR to 25th LRR containing ID. Interestingly, there are significant difference in conservation between the 1st to 10th LRR and 11th to 25th LRR among distinct plant species. The key amino acids of the 11th–25th LRRs were conserved and well matched in the extracellular domain of BRI1 from different plant species including *Arabidopsis thaliana, Brachypodium distachyon, Oryza sativa*, and *Zea mays* (Figure 3). However, the amino acid cannot well align in the 1st–10th LRRs of the extracellular domains due to insertion or deletion variation in BRI1 from different species (Figure 3). This indicates the first few LRRs after the signal peptide are less conserved among the BRI1 from distinct species. This high variation in the first 10 LRRs might indicate that the first 10 LRRs are expendable and have only minor or specific role for the receptors’ function. As such, its significance is difficult to uncover, constituting a reason that there is no missense mutants identified in this region so far. In the LRRs of extracellular domain, two mutants, *bri1-4* and *bri1-120*, occur at the 3rd and 13th LRRs, respectively. In *bri1-4*, a 10-bp segment is deleted, which results in premature termination of the translation resulting in truncated protein. Thus, the *bri1-4* gene is a null allele of *bri1*, whose mutant is comparable to the other null mutant phenotype (Noguchi et al., 1999). The *bri1-120* contains a substitution of Ser by Phe at the 399th amino acid in the 13th LRR. This leads to the conformational change affecting the receptor dimerization or reducing the ligand binding capacity (Shang et al., 2011). The finding of *bri1-120* showed that the LRR domain plays important role in BR perception. However, no information was known regarding the first few less conserved LRRs after the signal peptide in BRI1, which could also play a critical role in hormone perceiving and receptor activation. Recently, *bri1-706* mutants, carried a point mutation Ser-to-Phe in the 8th LRR of the extracellular portion of BRI1, were scanned *via* Targeted Induced Local Lesions IN Genomes (TILLING) analysis and waiting to be further analyzed (Sun et al., 2017).

In the present investigation, we reported the isolation of a weak *bri1* allele, *bri1-235*. This weak mutant generated by EMS mutagenesis had a single base change from C to T, which led to an amino acid substitution of Ser by Phe at the 156th position. This Ser residue is highly conserved in the 4th LRR of BRI1 (Figure 2). Like other canonical LRR protein, the concave surface of the BRI1 solenoid is composed of a parallel β sheet comprising 25 continuously running parallel β-strands. However, the BRI1 possesses parallel but distorted β-sheets following the inner β-structure in most of the repeats due to plant-specific consensus sequences. The loops of the convex outer surface are stabilized through disulfide bonds formed between consecutive repeats just except the 4th, 9th, and 13th LRR (She et al., 2011). As serine or threonine is able to form an additional hydrogen bond with other part of protein (Choe et al., 2005), the serine residue at the 4th LRR of BRI1 might participate in the formation of the hydrogen bond network, which stabilizes the assembly of repeating LRR motifs. Replacement of this solvent-exposed Ser residue by a bulky hydrophobic Phe reside in *bri1-235* mutant might cause a localized structural distortion. However, such a structural perturbation caused only mildly loss of the BRI1 function. When we overexpressed wild-type *bri1-235* in *bri1-235*, its phenotypes were rescued to almost the wild type (Figure 4). Furthermore, we found that *sbi1* (suppressor of *bri1*) also restored *bri1-235* to near the wild type (Figure 4). As previously reported, *sbi1* only acts on active BRI1 but not inactive BRI1 (Wu et al., 2011). Therefore, our results suggest that bri1-235 is still an active BR receptor. Apart from the serine at position 156, the other highly conserved residue in the 4th LRR is the asparagine (Asn) at position 154 (Figure 2B). According to N-glycosylation sites along the BRI1 superhelix from the C-terminus, this Asn residue is glycosylated and has an important role in structural stabilizes of BRI1 (Hothorn et al., 2011). Together Asn and Ser residues constitute a classic consensus N-glycosylation site (Asn-X-Ser). Mutation of the Ser at position 156 abrogates N-glycosylation at position 154, which may lead to local misfolding or trapping in ER quality control processes, and is the likely cause for the phenotype similar to previous findings for *bri1-5* and *bri1-9*, two best-characterized endoplasmic reticulum-localized *bri1* mutants. Thus, mutation of the Ser at position 156 critical for BRI1 folding may be involved to the glycosylation. Subcellular localization of proteins is a vital characteristic that depicts their functions in the cell. This is true for the hormonal receptors because they sense signaling molecules coming from outer space of the cells and transform this signal into the cell interior, thus triggering a precise biological process that affects primary cellular targets in the downstream. BRI1 was reported to undergo a highly conserved ER-mediated protein quality control (ERQC) mechanism before further trafficking to plasma membrane. Among identified BRI1 mutants, *bri1-5* and *bri1-9* are two typical examples whose proteins are retained in the endoplasmic reticulum. The dwarf phenotype of *bri1-9*, which carries a Ser-to-Phe mutation in the ligand binding domain of BRI1, is caused by endoplasmic reticulum retention of a structurally imperfect but functionally competent BR receptor *via* ERQC (Jin et al., 2007). In the endoplasmic reticulum, the stringency of the retention-based ERQC is reduced significantly due to mutations, and the bri1-9 protein was allowed to export to the cell surface for BR perception. Unlike *bri1-9*, *bri1-5* carries a Cys-to-Tyr mutation, which localizes in the amino-terminal pair cysteine before the beginning of the LRRs. The bri1-5 protein is also retained in the endoplasmic reticulum, involving three different retention mechanisms (Hong et al., 2008). Like *bri1-5* and *bri1-9*, the amino acid substitution in *bri1-235* occurs in the extracellular domain of BRI1. The susceptibility of bri1-235 to Endo H is similar to that of *bri1-5*, which retains in the ER (Figure 8). Moreover, the confocal analysis also shows that the bri1-235 is localized to ER instead of PM (Figure 8). Further investigation is required to find out whether *bri1-235* is involved in the disturbing of ERQC system or other retention mechanisms. Nevertheless, our study confirms that not only island and the subsequent 13th–25th LRR but also the first few LRRs after the signal peptide are critical for BRI1 folding.

## Conclusion

In summary, we demonstrated that the new *bri1* mutant, *bri1-235*, exhibited a weak morphological phenotype and a reduced sensitivity to exogenous BL. The point mutation is found in the first few less conserved LRRs after the signal peptide in BRI1. It was confirmed that the mutation resulted in an amino acid substitution from Ser to Phe at the 156th position of BRI1. Mutation occurred in the 1st–10th LRR regions are slightly conserved and are highly variable among different plant species. *bri1-235* can rescue *bri1-235* mutant to near wild type. Furthermore, *sbi1*, which acts on active BRI1, also restores the *bri1-235* to almost wild type. Therefore, the *bri1-235* is a new mutant having mislocalized functional BR receptor. Our findings likely provide insight into the crucial role of less conserved LRRs in BR receptor BRI1, shedding light on study of many other RLKs.

## Data Availability

All datasets generated for this study are included in the manuscript.

## Author Contributions

GL and QH conceived and designed the work. QH and SS performed the experiments and analyzed the data. HR helped with selecting this mutant. GL prepared the manuscript draft. KA, CB, and GW critically reviewed the manuscript. All authors read and approved the final manuscript.

### Conflict of Interest Statement

The authors declare that the research was conducted in the absence of any commercial or financial relationships that could be construed as a potential conflict of interest.

## Footnotes

^1^http://www.arabidopsis.org

^2^https://phytozome.jgi.doe.gov/pz/portal.html
